# A Unique Case of Hypothyroidism Causing Pancytopenia With Literature Review

**DOI:** 10.7759/cureus.27775

**Published:** 2022-08-08

**Authors:** Muzammil Khan, Zin Thawdar Oo, Zin M Htet, Vijaya Vudathaneni, Zinobia Khan

**Affiliations:** 1 Internal Medicine, North Central Bronx Hospital, Bronx, USA; 2 Internal Medicine, James J. Peters Department of Veterans Affairs Medical Center, Bronx, USA; 3 Critical Care Medicine, James J. Peters Department of Veterans Affairs Medical Center, Bronx, USA

**Keywords:** anemia, altered mental state, levothyroxine, pancytopenia, hypothyroidism

## Abstract

Severe endocrinopathies can lead to pancytopenia. Hypothyroidism can affect any organ system including hematopoietic, resulting mainly in a normochromic normocytic anemia. Rarely, in severe hypothyroidism, all hematopoietic cell lines can be affected, resulting in pancytopenia. We, hereby, would like to discuss a case of hypothyroidism with worsening pancytopenia, which gradually improved after repletion with levothyroxine. Although pancytopenia is not a common feature of hypothyroidism, this case highlights the importance of hypothyroidism being considered as one of the differentials.

## Introduction

Hypothyroidism can involve any organ system including the hematopoietic system, which mainly results in normochromic normocytic anemia, while other cell lines are rarely affected. Pancytopenia has been noted in several endocrinopathies. It can be associated with autoimmune thyroiditis and Graves’ disease; however, it is rarely reported in the setting of severe hypothyroidism. Here we present a case of worsening pancytopenia in a patient with hypothyroidism who had a subtherapeutic dose of levothyroxine, and improved cell count to baseline with an increasing dose.

## Case presentation

A 68-year-old woman with a history of hypertension and hypothyroidism was brought to the hospital after she was found wandering on the street. She was alert but incoherent during the conversation and complained of generalized body pain. She presented with hemoglobin (Hgb) of 9.0 g/dL (normal range for female 12.1-15.1 g/dL), white blood cell (WBC) 4850/uL (normal range 4500-11000/uL) and platelets of 102 x 10^9^/L (normal range 150-400 x 10^9^/L). Thyroid-stimulating hormone (TSH) on arrival was 54.650 U/L (normal range 0.470 - 6.900 U/L), thyroxine (T4) of < 0.10 ng/dL (normal range 0.750-2.000 ng/dL), and mild hyponatremia. She was started on her home medication of levothyroxine 75 mcg but her weight-based dose was calculated to be 119 mcg. She refused to take more than 75 mcg dose because she reported that this dose made her anxious with palpitations and hand tremors. All her cell lines were trending down while she was inpatient, with Hgb of 6.8 g/dL, WBC 2720/uL, and platelets of 47 x 10^9^/L. Anti-cytomegalovirus (CMV), anti-parvovirus antibody, and parasite screening were negative. Lactate dehydrogenase and haptoglobin were normal and not consistent with hemolysis. Vitamin B12 and folate were normal and reticulocytes showed good bone marrow response at 3.9%. Slight hypochromia of red blood cells and ovalocytes were seen on peripheral smear. Transferrin receptor 27.8 nmol/L (normal range 12.2-27.3 nmol/L), erythropoietin elevated at 106.3 mlU/mL (normal range 2.6-18.5 mlU/mL). Rheumatoid factor was also positive but anti-cyclic citrullinated peptide (CCP) returned negative. She was transfused one unit of packed red blood cells with an increase in Hgb to 8.1 g/dL. She eventually agreed to an increased dose of 88 mcg. TSH after increasing the dose was 57.970 U/L and T4 levels of 0.434 ng/dL. The increased dose caused all the cell lines to improve including Hgb of 8.0 g/dL, WBC 2960/uL, and platelets 96 x 10^9^/L. Once her mentation improved, she reported a prior history of pancytopenia, requiring transfusions but refused bone marrow biopsy on all occasions, including this time.

## Discussion

Hypothyroidism has been associated with anemia, most commonly normocytic but less likely to be of microcytic and macrocytic etiology. However, very few cases have been reported in the literature associating hypothyroidism with pancytopenia. Pancytopenia was noted in a patient with a myxedema coma, which responded with conversion to oral levothyroxine therapy.

The pathogenesis of pancytopenia in hypothyroidism is still unclear. While anemia, most likely normocytic, is secondary to the reduced erythrokinetic activity of bone marrow and decreased proliferative processing of the H-thymidine index. These changes in part were attributed to reduced oxygen requirement by tissues due to inhibited metabolic rate in hypothyroidism [1]. Anemia in hypothyroidism can be expected but the presence of pancytopenia is still unsettled. It is hypothesized that an autoimmune reaction can be the causative factor of pancytopenia in hypothyroidism, but its dissociation with thyroid peroxidase antibody makes it less likely. Marrow hypoplasia causing pancytopenia has also been suggested as a pathologic phenomenon [2,3]. The incidence of anemia is reported to be around 30% in hypothyroidism; in contrast, pancytopenia is very rare in hypothyroidism [2]. Prior studies have identified thyroid hormone as the endogenous signal responsible for the differentiation of cell lines.

In a case series by Laway et al. of Sheehan's syndrome with pancytopenia, it was suggested that thyroid hormone and cortisol deficiency were the main culprits of pancytopenia. However, many scientists and specialists have argued that thyroid hormone deficiency is the probable etiologic factor of pancytopenia [4]. Similarly, in other cases of hypopituitarism with hypothyroidism and hypocortisolism who were noted to have pancytopenia, there was swift normalization of cell lines after thyroid hormone replacement [5-7]. Here we have given a brief account of reported cases of pancytopenia in the setting of hypothyroidism with their presentation and associated symptoms (Table 1).

**Table 1:** Reported cases of pancytopenia in the setting of hypothyroidism with their presentations and associated symptoms M: male; F: female; g/dL: grams per deciliter; uL: microliter; L: liter; Hbg: hemoglobin; WBC: white blood cell; IV: intravenous

Author	Gender	Cell Counts	Reference values	Treatment	Follow-up
Current case	F	Hgb 9.0 g/dL, WBC 4850/uL, Platelets 102 x 10^9^/L	Hbg 12.1-15.1 g/dL (female), WBC 4500-11000/uL, Platelets 150-400 x 10^9^/L	Oral levothyroxine	Improvement of cell lines within one week of treatment
Rathi et al. [2]	F	Hgb 10.2 g/dL, WBC 2900/uL, Platelets 127 x 10^9^/L	Hbg 12.1-15.1 g/dL (female), WBC 4500-11000/uL, Platelets 150-400 x 10^9^/L	Oral levothyroxine	Resolved pancytopenia after discharge follow-up
Song et al. [3]	F	Hgb 8.2 g/dL, WBC 1600/uL, Platelets 35 x 10^9^/L	Hbg 12.1-15.1 g/dL (female), WBC 4500-11000/uL, Platelets 150-400 x 10^9^/L	Oral levothyroxine, hydrocortisone, and triiodothyronine	Resolution of white cell count and platelet count in six weeks of discharge
Samanta et al. [8]	M	Hgb 6.8 g/dL, WBC 2200/uL, Platelets 110 x 10^9^/L	Hbg 13.8-17.2 g/dL (male), WBC 4500-11000/uL, Platelets 150-400 x 10^9^/L	Oral levothyroxine	Improvement of pancytopenia within two weeks and resolution in six weeks
Shaaban et al. [9]	F	Hgb 8.4 g/dL, WBC 2500/uL, Platelets 95 x 10^9^/L	Hbg 12.1-15.1 g/dL (female), WBC 4500-11000/uL, Platelets 150-400 x 10^9^/L	IV and oral levothyroxine	Improvement of cell lines within one week of treatment
Tsoukas et al. [10]	F	Hgb 8.5 g/dL, WBC 3990/uL, Platelets 27 x 10^9^/L	Hbg 12.1-15.1 g/dL (female), WBC 4500-11000/uL, Platelets 150-400 x 10^9^/L	IV and oral levothyroxine, IV hydrocortisone	Pancytopenia subsided four weeks after discharge
McMahon et al. [11]	F	Hgb 6.9 g/dL, WBC 3800/uL, Platelets 158 x 10^9^/L	Hbg 12.1-15.1 g/dL (female), WBC 4500-11000/uL, Platelets 150-400 x 10^9^/L	Oral levothyroxine + vitamin B12	Pancytopenia resolved within two months with treatment

Pancytopenia in the vitamin or nutritional deficiency setting has been reported abundantly in the literature review. It was considered in our patient since these are the most commonly encountered reversible causes. Folate and vitamin B12 deficiency are the most common culprits, causing arrested development of cell lines causing inhibited cell counts [12,13]. The effects become more prominent when it is an elderly patient. This etiology can be reversed with adequate replenishment of the deficient vitamin. However, vitamin B12 and folate levels were normal in our patient.

Our patient also presented other signs and symptoms of hypothyroidism, including altered mental status, hyponatremia, and anemia. An extensive workup was done to rule out other causes of pancytopenia and all returned negative. Her mental status and cell lines returned to baseline with repletion with levothyroxine. Biopsy was considered and advised but she refused multiple times. On further inquiry, she reported a long-term history of hypothyroidism and non-compliance with medications. The patient also reported a prior history of pancytopenia, requiring transfusions but refused bone marrow biopsy on all occasions. Just like the majority of reported cases, our patient was also female.

## Conclusions

We recommend that severe hypothyroidism be considered as one of the differentials for pancytopenia. Although anemia and leukopenia are common in uncontrolled hypothyroidism, inhibition of all cell lines including platelet count is rarely seen, which makes our case more interesting and unusual. Our patient did not develop a coma and her initial altered mental status improved with adequate repletion of levothyroxine.
